# Expression of cancer testis antigens and its correlation with clinicopathological parameters in hepatocellular carcinoma

**DOI:** 10.1186/2051-1426-1-S1-P250

**Published:** 2013-11-07

**Authors:** Meng Wang, Xin-feng Chen, Zhi-qin Li, Zhen Zhang, Dong-li Yue, Yan-hong Liu, Dong Jiang, Yu-ling Sun, Yong-fu Zhao, Shu-huan Wu, Jian-sheng Li, Yi Zhang

**Affiliations:** 1 Department of Gastroenterology, the First Affiliated Hospital of Zhengzhou University, Zhengzhou, China; 2 Biotherapy Center, the First Affiliated Hospital of Zhengzhou University, Zhengzhou, China; 3 Department of Infectious Diseases, the First Affiliated Hospital of Zhengzhou University, Zhengzhou, China; 4 Department of Hepatobiliary Surgery, the First Affiliated Hospital of Zhengzhou University, Zhengzhou, China; 5 Department of Bioengineering, the First Affiliated Hospital of Zhengzhou University, Zhengzhou, China; 6 Department of Oncology, the First Affiliated Hospital of Zhengzhou University, Zhengzhou, China

## Background

Many therapies such as surgery, chemical or physical approaches have been used for treatment of HCC; however, the outcome is still poor. Cancer immunotherapy is considered to be one of the promising strategies in recent years.

## Methods

Expression of CTAs genes including MAGE A3, MAGE A4, MAGE C2 and NY-ESO-1 genes was detected with reverse transcription polymerase chain reaction (RT-PCR) in HCC tissues and corresponding adjacent non-cancerous tissues from 71 HCC patients.

## Results

87.3% of HCC tumor tissue samples expressed at least 1 CTAs. HCC adjacent non-cancerous tissues did not express CTAs. 78.9% tumor tissue samples expressed MAGE-A3 mRNA, 33.8% samples expressed MAGE-A4 mRNA, 74.6% samples expressed MAGE-C2 mRNA and 14.1% samples expressed NY-ESO-1 mRNA. The expression of CTAs showed correlation with Ki67(r=0.27, P=0.02) and tumor stages (r=0.31, P=0.01), no correlation with clinical parameters such as age, gender, ALT, HLA-A2 positive, CA125, CA199, HBV or HCV infection and tumor size (P>0.05). The expression of MAGE-A3 showed correlation with the high expressions of CEA in serum (r=0.30, P=0.03), AFP in serum (r=0.26, P=0.03) and lymph node metastases (r=0.30, P=0.01).

## Conclusion

Our findings demonstrate the cancer testis expression of CTAs genes show correlations with tumor stages and proliferation and may represent useful targets for tumor specific immunotherapy in HCC patients.

**Table 1:** Clinicopathological characteristics of the HCC patients

Characteristics	No.patients	%
Gender	71	
Male	54	76.1
Female	17	23.9
Age(y)		
<55	47	66.2
≥55	24	33.8
ALT(IU/ml)		
<80	61	85.9
≥80	10	14.1
HLA A2		
Positive	41	57.7
Negative	30	42.3
AFP(ng/ml)		
≤10	30	42.3
>10	41	57.7
Tumor size(cm)		
≤5	43	60.6
>5	28	39.4
TNM classification		
I or II	34	47.9
III or IV	37	52.1
Etiology		
HBV	45	63.4
HCV	6	8.5
No	20	28.1
Ki67(%)		
≤40	30	42.3
>40	41	57.7
CTAs		
Positive	62	87.3
Negative	9	12.7
Average age(year)	53.7±11.5	

